# Synthesis of Highly Monodisperse Nickel and Nickel Phosphide Nanoparticles

**DOI:** 10.3390/nano12183198

**Published:** 2022-09-14

**Authors:** Hyungjin Cho, Nohyun Lee, Byung Hyo Kim

**Affiliations:** 1 Department of Organic Materials and Fiber Engineering, Soongsil University, Seoul 06978, Korea; 2 School of Advanced Materials Engineering, Kookmin University, Seoul 02707, Korea; 3 Department of Green Chemistry and Materials Engineering, Soongsil University, Seoul 06978, Korea

**Keywords:** nanoparticles, nickel, nickel phosphide, his-tagged protein

## Abstract

Nickel and nickel phosphide nanoparticles are highly useful in various fields, owing to their catalytic and magnetic properties. Although several synthetic protocols to produce nickel and nickel phosphide nanoparticles have been previously proposed, controllable synthesis of nanoparticles using these methods is challenging. Herein, we synthesized highly monodisperse nickel and nickel phosphide nanoparticles via thermal decomposition of nickel–oleylamine–phosphine complexes in organic solvents. The size and composition of the nickel and nickel phosphide nanoparticles were easily controlled by changing the aging temperature, precursor concentration, and phosphine surfactant type. Large-sized monodisperse nickel nanoparticles obtained using our method were successfully applied for the purification of histidine-tagged proteins.

## 1. Introduction

The synthesis of magnetic nanoparticles is important because of their broad applications in magnetic resonance imaging, drug delivery, magnetic separation, catalysis, and magnetic storage devices [1,2,3,4,5]. As physical properties of nanoparticles are affected by their size and shape, synthetic methods allowing the control of particle size, size distribution, and particle shape are strongly desired [6,7,8]. Nickel and nickel oxide nanoparticles are widely used as catalysts [9,10], in magnetic separation of histidine-tagged proteins [11], in resistive random access memory [12], and as superconductors [13]. Nickel nanoparticles have been previously synthesized using sonochemistry [14], reduction [15,16,17], decomposition of organometallic compounds or metal salts [18,19], and electrochemical methods [20]. However, nanoparticles synthesized using these methods are polydisperse, have low crystallinity, and are prone to aggregation. Monodisperse nickel nanoparticles have also been synthesized using the thermal decomposition of organometallic complexes in organic solvents. However, the diameter of the monodisperse nickel nanoparticles synthesized via the abovementioned procedures is usually smaller than 10 nm [21,22,23,24], and small nanoparticles are fully oxidized to antiferromagnetic nickel oxide nanoparticles with a low magnetic moment [24]. Nickel phosphide nanoparticles are also important materials for catalysis, supercapacitors, and electrocatalysis [25,26,27,28].

Metal phosphide nanoparticles have been previously synthesized using phosphine [29,30,31,32,33], phosphate salts [34], and pure phosphorous as phosphorous sources [35,36]. In recent works, nickel nanoparticles have often been used as scaffolds for producing nickel phosphide nanoparticles [37,38]. The reported procedures, however, are tedious, and the resulting nanoparticles are polydisperse. 

A simple method for the synthesis of monodisperse nickel and nickel phosphide nanoparticles is presented in this report. The resulting nickel nanoparticles are highly monodisperse and sufficiently large to maintain a pure nickel core even after oxidation in air. Moreover, both nickel and nickel phosphide nanoparticles can be obtained using a single synthetic scheme reported here, only at different aging temperatures.

## 2. Materials and Methods

### 2.1. Materials

Nickel(II) acetylacetonate (Ni(acac)_2_, 90%), octyl ether (99%), trioctyl phosphine (TOP, 90%), 1-octadecene (90%), and oleic acid (90%) were purchased from Sigma-Aldrich (St. Louis, MO, USA). Tributyl phosphine (TBP) was purchased from Fluka (Charlotte, NC, USA). Oleyl amine was purchased from Acros. All chemicals were used without further purification. 

Histidine-tagged green fluorescent protein (GFP) and PE-Cy5-conjugated normal mouse IgG1 were purchased from Santa Cruz Biotechnology (Dallas, TX, USA) and Upstate, respectively.

### 2.2. Methods

#### 2.2.1. Synthesis of 9 nm Nickel Nanoparticles

Ni–oleylamine complex was prepared by reacting 1.55 g of oleylamine with 0.257 g of Ni(acac)_2_ at 100 °C for 30 min under an inert atmosphere. Dioctyl ether (6 mL) was injected into the Ni–oleylamine complex, and the solution was degassed for 1 h at 100 °C under vacuum. TBP (0.3 mL) was injected into the nickel complex solution under an argon atmosphere. The solution was heated to 250 °C with a heating rate of 2 °C/min and aged at the same temperature for 30 min. At approximately 200 °C, the color of the solution started to change from bluish-green to black, indicating the formation of nickel nanoparticles. After aging, the product was rapidly cooled to 70 °C. Oleic acid (2 mL) was added to the product under argon flow to stabilize the nanoparticle dispersion. Nanoparticles were precipitated by adding a 1:2 mixture of excess ethanol and acetone. The nanoparticles were dispersed in hydrophobic solvents, such as hexane or chloroform.

#### 2.2.2. Synthesis of 13 nm Nickel Nanoparticles

Dioctyl ether (5 mL) was injected into the Ni–oleylamine complex, and the solution was degassed for 1 h at 100 °C under vacuum. TBP (0.3 mL) was injected into the nickel complex solution under an argon atmosphere. The solution was then heated to 250 °C with a heating rate of 2 °C/min and aged at the same temperature for 30 min.

#### 2.2.3. Synthesis of Ni_12_P_5_ Nanoparticles

Dioctyl ether (5 mL) was injected into the Ni–oleylamine complex, and the solution was degassed for 1 h at 100 °C under vacuum. TBP (0.3 mL) was injected into the nickel complex solution under an argon atmosphere. The solution was then heated to 280 °C with a heating rate of 2 °C/min and aged at the same temperature for 30 min.

#### 2.2.4. Ligand Exchange of Nickel/Nickel Oxide (Ni/NiO) Nanoparticles

The nanoparticles (200 mg) oxidized in air were dispersed in an imidazole solution in chloroform (0.1 g/mL, 5 mL) and stirred for 6 h. The ligand-exchanged nanoparticles were collected by centrifugation after adding n-hexane. 

#### 2.2.5. Reaction of Ni/NiO Nanoparticles with Proteins

The imidazol-stabilized oxidized Ni nanoparticles (60 μg) were mixed with 250 μL of protein solution with a concentration of 30 μg/mL. The protein solutions were prepared by dissolving histidine-tagged GFP or PE-Cy5-conjugated normal mouse IgG1 antibodies in PBS. The mixture containing Ni/NiO nanoparticles and fluorescent proteins was incubated by shaking for 30 min. The Ni/NiO nanoparticles and bound proteins were purified using a magnet. The purified particles and proteins were redispersed in 250 μL of imidazole solution (0.1 g/mL). The proteins bound to the Ni/NiO nanoparticles were released by shaking for 30 min.

#### 2.2.6. Characterization of Materials

Transmission electron microscopy (TEM) and high-resolution TEM (HRTEM) images were obtained by JEOL EM-2010 and JEOL EM-2100F at an accelerating voltage of 200 kV. X-ray diffraction (XRD) patterns were acquired using a Rigaku Dmax 2500 diffractometer using monochromatized Cu-Kα radiation at 40 kV and 200 mA. The hydrodynamic size of the ligand-exchanged nanoparticles was characterized by Otsuka DLS-8000. The magnetization measurements were performed using an MPMS 5XL Quantum Design superconducting quantum interference device (SQUID) magnetometer. Photoluminescence spectra were collected on a Perkin-Elmer LS 50 B spectrometer at excitation wavelengths of 400 nm (for GFP) and 630 nm (for Cy5).

## 3. Results and Discussion

### Analysis of Ni Nanoparticles

**Figure 1:**
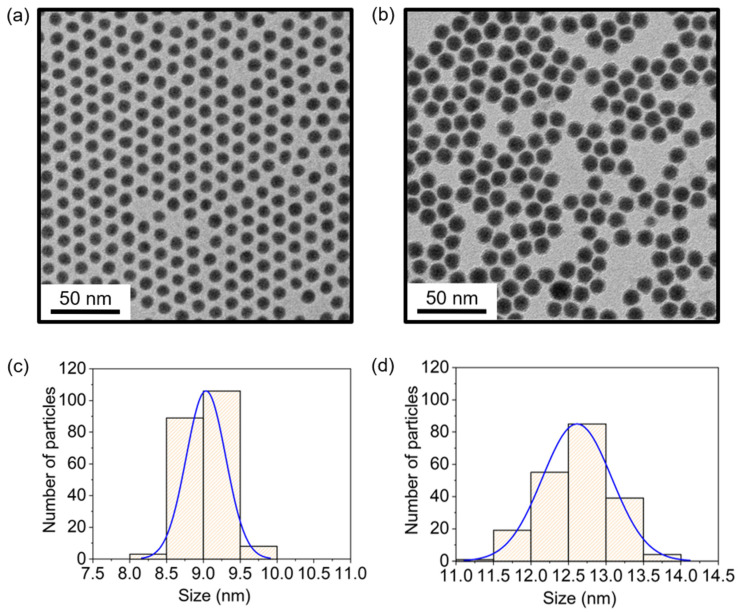
TEM images of (**a**) 9 nm and (**b**) 13 nm nickel nanoparticles obtained at precursor concentrations of 167 and 200 mM, respectively. Scale bars are 50 nm. The corresponding size histograms are displayed in (**c**,**d**). The average diameters of the nanoparticles were (**c**) 9.03 nm and (**d**) 12.61 nm. The standard deviations were (**c**) 2.97% (0.268 nm) and (**d**) 3.67% (0.463 nm). Blue lines are Gaussian fitted lines. To ensure the accuracy of size measurement, we measured the sizes of 208 individual particles for each batch.

TEM images of Ni nanoparticles synthesized using TBP as a surfactant are shown in Figure 1. The images show highly monodisperse Ni nanoparticles obtained without any size selection. The large-area two-dimensional assembly of nanoparticles confirmed their monodispersity, as shown in Figure S1. The particle sizes were controlled by regulating the precursor concentration: nanoparticles with diameters of 9 and 13 nm (Figure 1a,b) were synthesized at precursor concentrations of 167 and 200 mM, respectively. Size distribution histograms proved that the particles were very monodisperse, with the coefficient of variation for size not exceeding 4% (Figure 1c,d).

The HRTEM image of the 13 nm nanoparticles observed one month after the synthesis revealed the polycrystalline structure of the nickel core and nickel oxide shell (Figure S2). With continuous exposure to air, nickel atoms on the surface of the nanoparticles are oxidized and form a ~3 nm thick nickel oxide layer, owing to the limited diffusion depth of oxygen [39]. While large nickel particles are partially oxidized, nanoparticles smaller than 7 nm are completely oxidized to nickel oxide nanoparticles [40,41]. The HRTEM image showed voids at the interface between the nickel and nickel oxide layers and bridges in the voids. The voids and bridges between the two components are typical features of the nanoscale Kirkendall effect. The Kirkendall effect is an atomic transfer phenomenon at the interface of a diffusion couple [9,42]. When atomic diffusion occurs through vacancy exchange, the outward flow of fast-moving cations and inward flow of slow-moving oxide anions produce a metal/metal oxide core/shell structure with voids at the interface between the metal and metal oxide. The Kirkendall effect can occur at room temperature if atomic diffusivity is low for a sufficiently long time. Upon exposure to air for a month, the 13 nm nickel nanoparticles changed to 16 nm Ni/NiO nanoparticles, as shown in the HRTEM image (Figure S2). This peculiar change may also be the result of the Kirkendall effect. The core nickel cations moved faster than the outer oxide anions. As a result, a nickel oxide layer was formed, and the size of the nanoparticles increased. 

**Figure 2:**
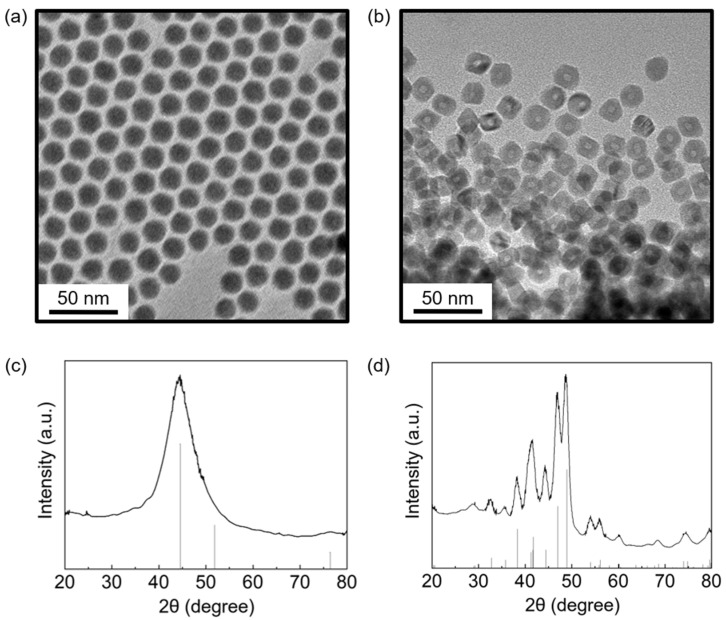
TEM images of (**a**) Ni and (**b**) Ni_12_P_5_ synthesized at (**a**) 240 and (**b**) 280 °C (precursor concentration = 200 mM, surfactant: TBP, solvent: dioctyl ether) and XRD spectra of (**c**) Ni (a, JCPDS number 87-0712) and (**d**) Ni_12_P_5_ (b, JCPDS number 74-1381) nanoparticles.

The crystal structures of the nickel nanoparticles were characterized using XRD. The large and broad (111) peak at 44° and the small (220) peak at 77° in the XRD pattern in Figure 2c indicate that the particles consisted of face-centered-cubic (fcc) Ni. The broad peaks imply that the particles had a small grain size. The grain size calculated using the Scherrer equation was 2.2 nm [43], which was much smaller than the core size measured by TEM (~9 nm), indicating that the core material had a polycrystalline morphology. The polycrystalline nature of the obtained nickel nanoparticles was also identified using HRTEM (Figure S2). Nickel oxide peaks were absent in the XRD pattern because the metal oxide shell formed through the Kirkendall effect has amorphous morphology [44]. The oxidation of nickel was confirmed using X-ray photoelectron spectroscopy (XPS). The XPS data exhibited two peaks related to Ni^2+^ ions on the surface (Figure S3).

Phosphine surfactants, TBP and TOP, were used to synthesize nickel nanoparticles. The use of TOP as a surfactant in a 200 mM precursor produced nickel nanoparticles with a size of 10 nm. At the same time, 13 nm nickel nanoparticles were synthesized using TBP as a surfactant (Figure S4). During aging, bulky TOP molecules limit the growth of nanoparticles, resulting in the formation of small particles [22].

The chemical composition of nanoparticles was controlled by varying the aging temperature. The XRD patterns of the nanoparticles aged at 240 and 280 °C are shown in Figure 2c,d. The XRD patterns demonstrated that the nanoparticles aged at 280 °C consisted of Ni_12_P_5_ with (440), (312), (510), and (004) peaks and that those aged at 240 °C were fcc Ni particles. Ni_12_P_5_ particles had hollow structures (Figure 2b and Figure S5); the phosphorus source for their formation was trialkyl phosphine. Phosphine has been previously used either as a phosphorus source for the synthesis of metal phosphide nanoparticles [33] or as a surfactant for metal nanoparticles [45,46]. In most cases, when trialkyl phosphine was used as a phosphorus source, nanoparticles are synthesized at temperatures higher than 280 °C. Above 280 °C, the carbon–phosphorus bonds in trialkyl phosphines are dissociated owing to the high thermal energy. Free phosphorus atoms participate in the formation of nanoparticles, resulting in the formation of metal phosphides. Moreover, the surface metal atoms of the nanoparticles can act as catalysts to break carbon–phosphorus bonds and promote phosphide formation reactions [32]. At aging temperatures below 250 °C, trialkyl phosphines act as surfactants [22,45]. Below 250 °C, carbon–phosphorus bonds in trialkyl phosphines remain intact because of low thermal energy. Therefore, at low temperatures, nickel nanoparticles were obtained, whereas at high temperatures, nickel phosphide nanoparticles were synthesized, as shown in the XRD patterns. The differential scanning calorimetry (DSC) data for TBP support this explanation (Figure S6). Two endothermic peaks that indicate the dissociation of the carbon–phosphorus bond were observed at 255 and 296 °C in the DSC results.

**Figure 3:**
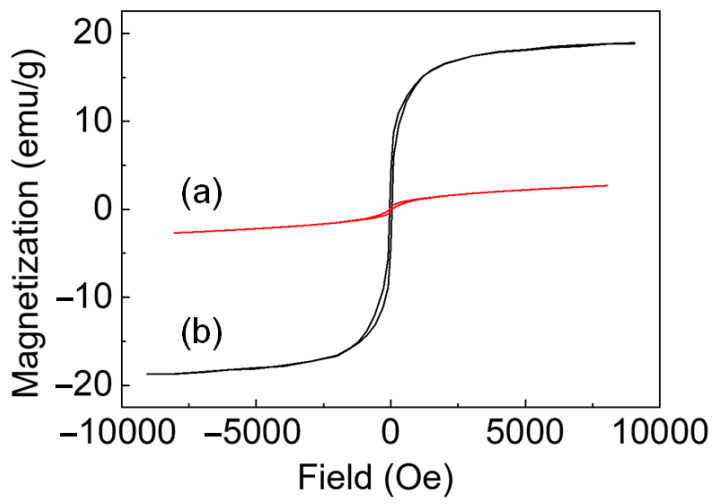
Magnetization (*M*)–magnetic field (*H*) curve for Ni nanoparticles measured by SQUID at 5 K for (a) 13 nm and (b) 18 nm Ni nanoparticles.

The magnetic properties of nickel nanoparticles are dependent on their size. The magnetization–magnetic field curve of 13 nm Ni nanoparticles measured using SQUID showed that the saturation magnetization of the nanoparticles at 8000 Oe was 2.8 emu/g (Figure 3), which is much smaller than the saturation magnetization of bulk nickel (56 emu/g). The small magnetization of nickel nanoparticles is also reported in the literature on the synthesis of nickel nanoparticles [14,47,48,49]. This result can be explained by several reasons. First, a small volume of the magnetic region may cause small magnetization. Ni nanoparticles changed into a Ni/NiO core/shell structure via surface oxidation (Figure S2). Ni/NiO nanoparticles mostly consist of an antiferromagnetic nickel oxide layer, which has much weaker magnetic properties than Ni. Second, the polycrystalline morphology may reduce magnetization [14,49]. Incomplete coordination of nickel atoms on the surface or grain boundaries of particles leads to spin disordering. This disorder has been interpreted as the cause of the small saturation magnetization. Third, phosphorus impurities may be responsible for reduced magnetism. As discussed in the previous section, nickel phosphide nanoparticles were synthesized through the breakage of the carbon–phosphorus bonds in trialkyl phosphine at temperatures higher than 280 °C. At the same time, bond breakage can still occur below 280 °C because of the distribution of thermal energy. Therefore, we assumed that the phosphorus atoms derived from phosphine acted as impurities. The exchange coupling can be interrupted by impurities, resulting in decreased magnetization [50].

To obtain nanoparticles with high magnetism, both the crystallinity and size of the particles should be increased while reducing the amount of impurities. When a smaller amount of phosphine was used, larger particles with high crystallinity and fewer impurities were obtained. Because phosphine molecules can induce the decomposition of the Ni–oleylamine complex [21], the use of a small amount of phosphine leads to the slow nucleation of nickel. The smaller number of nuclei formed during slow nucleation grew to larger particles because of the higher number of atoms per nuclei [51]. At the same time, phosphorus impurities expand the lattice parameter. When more phosphine was used, the position of the Ni(111) peak in the XRD patterns shifted to a lower angle, and the calculated lattice parameter increased. The relationship between the amount of phosphine and the lattice parameter is shown in Figure S7 and Table S1. The particles synthesized with the lowest amount of impurities (precursor: phosphine = 1:0.6) were the largest (18 nm). The 18 nm nickel nanoparticles were highly attracted to magnets and showed a saturation magnetization of 19 emu/g and a blocking temperature of 167 K (Figure 3, Figures S8 and S9). Previous literature reported that carbon impurity can be inserted into Ni at the aging temperature we used [52]. 

**Figure 4:**
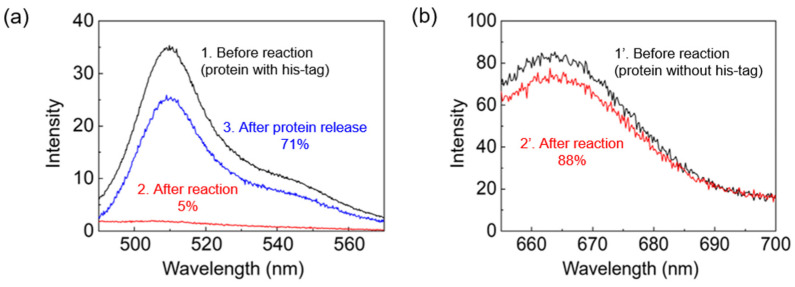
(**a**) Fluorescence spectra for the solutions of histidine-tagged GFP (1) before and (2) after the treatment with Ni/NiO nanoparticles and (3) after the treatment of protein-bound Ni/NiO nanoparticles with imidazole solution. (**b**) Fluorescence spectra for the solutions of normal mouse IgG1 labeled with Cy5 (1′) before and (2′) after the treatment with Ni/NiO nanoparticles.

Nickel nanoparticles were applied for the magnetic separation of histidine-tagged proteins, which requires Ni^2+^ cations. Ni^2+^ ions have a high affinity for polyhistidine and are exposed at the surface of the nickel nanoparticles after the oxidation of nickel to nickel oxide under air exposure [11]. For the biomedical protein separation, we transformed the hydrophobic nickel nanoparticles into hydrophilic particles to ensure their dispersion in the water phase using ligand exchange (Figure S10). For the purpose of protein separation by the Ni/NiO nanoparticles, we used polyhistidine-tagged GFP because the fluorescent property of the protein enables visualization and characterization of the successful separation of the protein. In a typical experiment, nickel nanoparticles were incubated with histidine-tagged GFP for 30 min and then separated from the supernatant through the application of the magnetic field. The fluorescence emission of the supernatant was significantly lower (~5%) than that of the initial solution (Figure 4). The histidine-tagged GFP-bound nanoparticles were then incubated with imidazole solution to release GFP proteins from the nanoparticles. The fluorescence emission recovered to 71% of the initial solution after the release of bound GFP proteins. This result indicated the efficient binding and separation of histidine-tagged GFP by Ni/NiO nanoparticles. Although the nanoparticles are capped with ligands, the purification of histidine-tagged proteins is activated by the partially exposed Ni^2+^ ions on the surfaces. It has been known that the nanoparticle surface is not fully coordinated with ligands due to the geometric factor, especially for bulky ligands such as imidazole. In addition, adsorption and desorption of ligands on the nanoparticle surface are reversible reactions; thus, Ni^2+^ ions can be exposed during adsorption and desorption. As a control experiment, we conducted an identical experiment using normal mouse IgG1 labeled with red-emitting Cy5 without a polyhistidine residue instead of histidine-tagged GFP. For the conventional dye-labeled protein, we observed only a 12% decrease in the fluorescence intensity, indicating a small extent of nonspecific protein binding. 

## 4. Conclusions

We synthesized highly monodisperse nickel nanoparticles, which were transformed into a Ni/NiO core/shell structure via oxidation of the surface nickel atoms. We also synthesized nickel phosphide nanoparticles using the same procedure but at a higher aging temperature. At a higher temperature, the carbon–phosphorus bonds in alkyl phosphines break, generating free phosphorus atoms to participate in the formation of nickel phosphide nanoparticles. The resulting nickel nanoparticles can be used for the magnetic separation of histidine-tagged proteins.

Our synthetic system is simple and highly reproducible. As our synthesis does not require a hot injection step, it can be used to synthesize nickel particles on a large scale. We were able to obtain 1.4 g of nickel nanoparticles in a single batch using 10 times more reagent than reported here.

## Data Availability

The data presented in this study are available on request from the corresponding author.

## Supplementary Materials

The following supporting information can be downloaded at: https://www.mdpi.com/article/10.3390/nano12183198/s1, Figure S1: Large-area TEM image of 9 nm nickel nanoparticles.; Figure S2: TEM image of polycrystalline Ni/NiO core/shell nanoparticles after oxidation of nickel nanoparticles.; Figure S3. X-ray photoelectron spectroscopy (XPS) spectrum of 9 nm nickel nanoparticles.; Figure S4. TEM images of (a) 10 nm and (b) 13 nm Ni nanoparticles, synthesized using TOP and TBP as surfactants, respectively.; Figure S5. HRTEM images of hollow Ni_12_P_5_ nanoparticles synthesized at 280 °C.; Figure S6. DSC data of TBP.; Figure S7. XRD patterns of (a) 13 nm, (b) 15 nm, (c) 17 nm, and (d) 18 nm Ni nanoparticles synthesized when the precursor to phosphine ratio was 1:1.2, 1:1.0, 1:0.8, and 1:0.60, respectively.; Table S1. Lattice parameters of Ni nanoparticles obtained by varying precursor to phosphine ratio.; Figure S8. Temperature dependence of magnetization measured with 100 Oe for (a) 13 nm and (b) 18 nm Ni nanoparticles.; Figure S9. Magnetization–magnetic field curves of Ni nanoparticles measured by SQUID at 300 K for (a) 18 nm and (b) 13 nm Ni particles.; Figure S10. The hydrodynamic size distribution of nickel nanoparticles after the ligand exchange process as measured by DLS.
