# Supplementary material for: Synthesis of Highly Monodisperse Nickel and Nickel Phosphide Nanoparticles
Source: Nanomaterials (Basel). 2022 Sep 14;12(18):3198. doi: 10.3390/nano12183198 (PMC9503933; doi:10.3390/nano12183198)
Supplement: Supplementary file 1 [file nanomaterials-12-03198-s001.zip › nanomaterials-1893766-supplementary.pdf]

Supplementary Materials for:

Synthesis of Highly Monodisperse Nickel and Nickel Phosphide Nanoparticles

Hyungjin Cho, Nohyun Lee and Byung Hyo Kim *

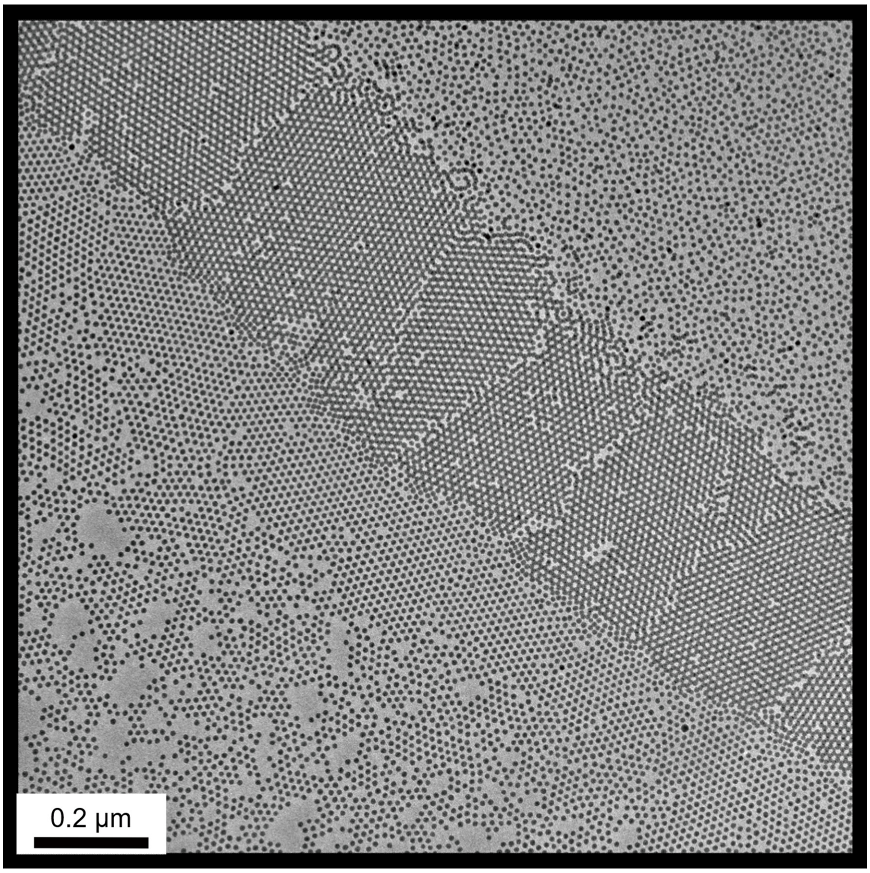

Figure S1. Large area image of 9 nm Ni nanoparticles. Scale bar is 0.2 µm.

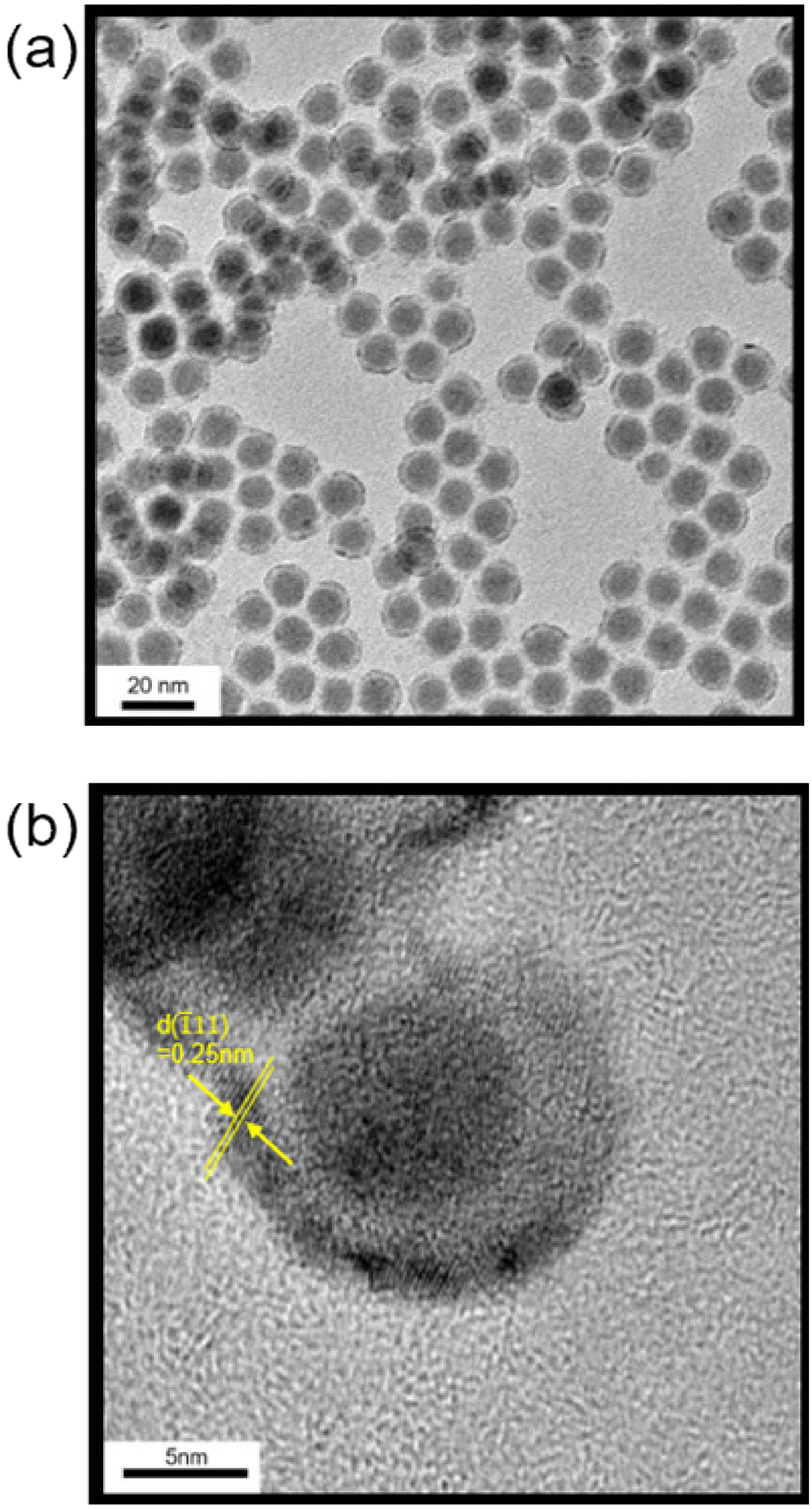

Figure S2. The polycrystalline structure of Ni core and NiO shell image shows that there is Kirkendall reaction on the Ni particles. Being exposed to air for a month, 13 nm Ni nanoparticles changed to 16 nm Ni/NiO nanoparticles. Scale bar is (**a**) 20 nm, (**b**) 5 nm.

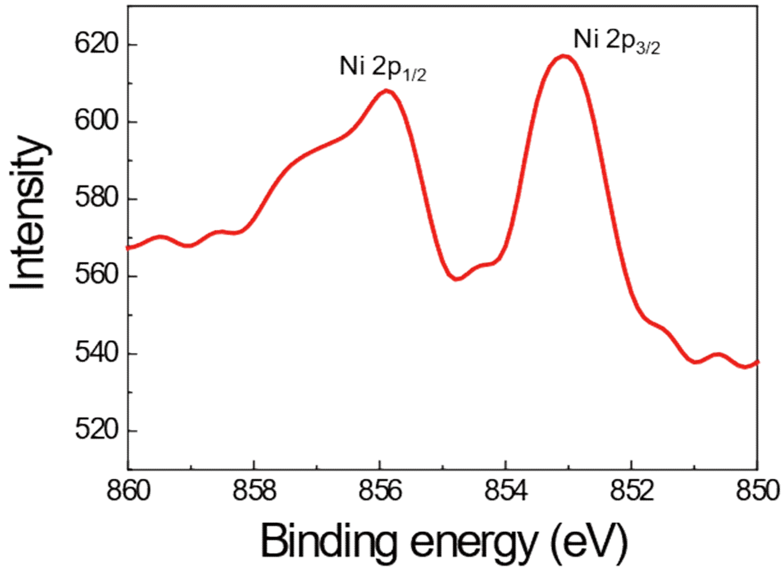

Figure S3. XPS spectrum in the region of Ni 2p core level obtained from 9 nm Ni nanoparticles. 853 eV peak represent Ni(0) metal, on the other hand, 856 eV peak is more oxidized Ni(II) peak. XPS data exhibit that there are Ni^{2+} ions on the surfaces of particles.

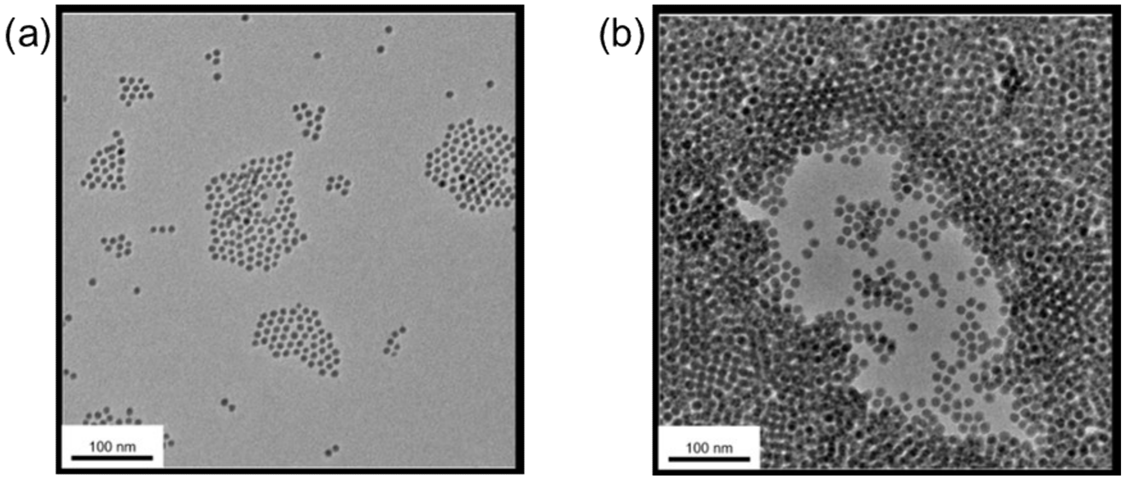

Figure S4. TEM images of (**a**) 10 nm and (**b**) 13 nm Ni nanoparticles, synthesized by using TOP and TBP (as surfactant), respectively. (Precursor concentration = 200 mM, solvent: dioctyl ether, aging temperature: 250 °C) Scale bar is 100 nm

(**a**)

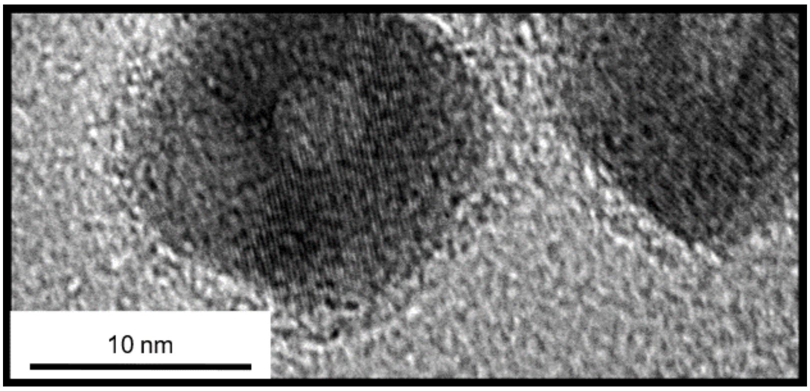

(**b**)

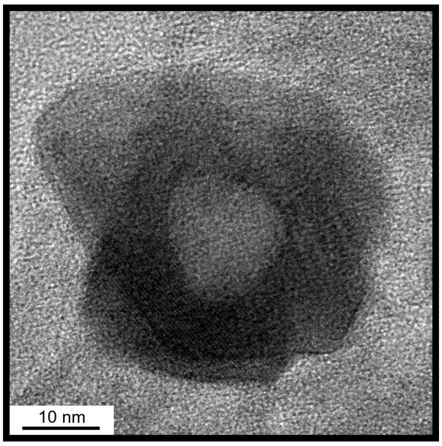

Figure S5. HRTEM images of hollow $Ni_{12}P_5$ nanoparticles synthesized at 280 °C. (**a**) Precursor concentration = 200 mM, surfactant: TBP, solvent: dioctyl ether. (**b**) Precursor concentration = 200 mM, surfactant: TOP, solvent: dioctyl ether. Due to the particle overlap, the size of particles looks large.

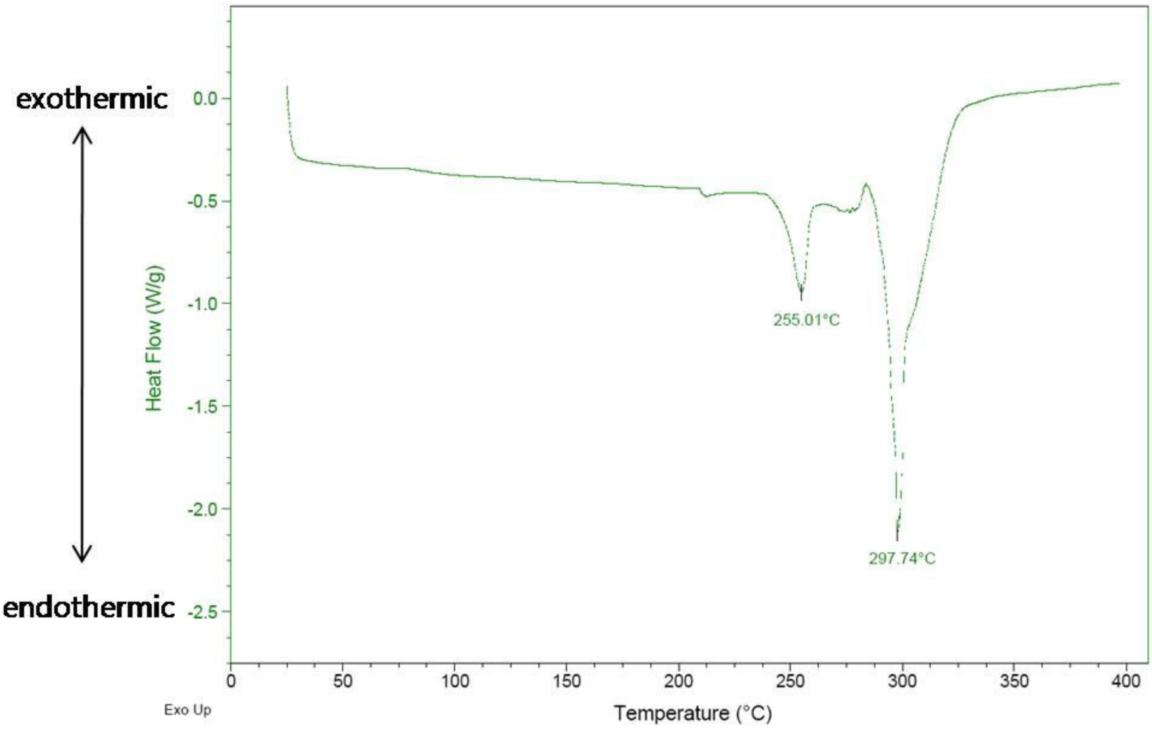

Figure S6. DSC data of TBP. Endothermic peaks that indicate the dissociation of carbon-phosphorus bond lie at 255 °C and 298 °C.

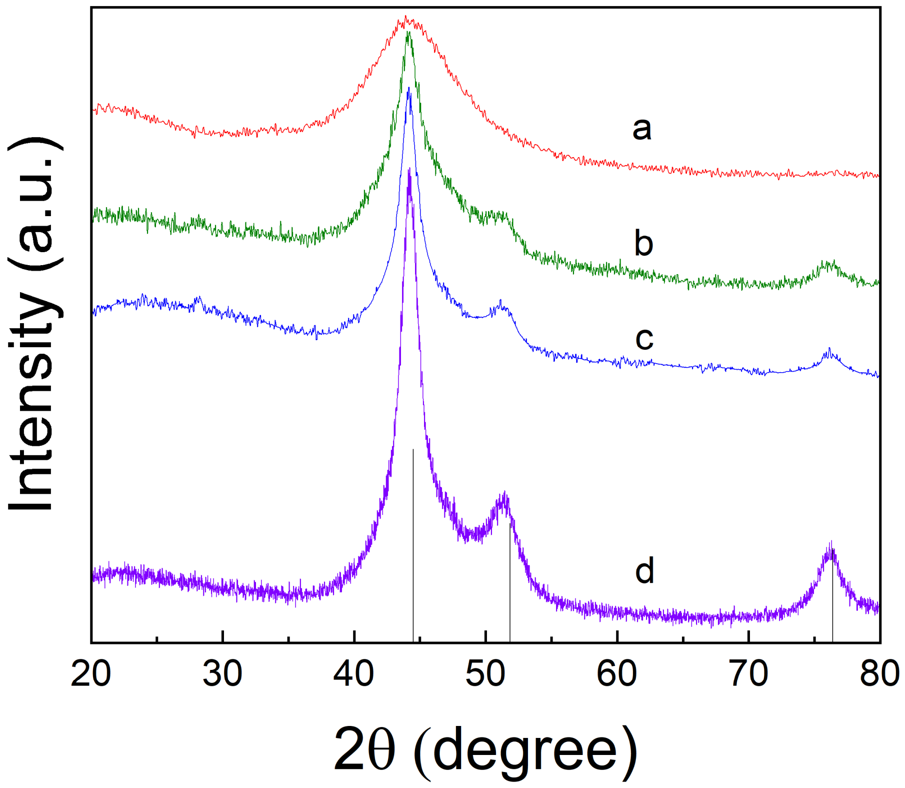

Figure S7. XRD patterns of (**a**) 13 nm, (**b**) 15 nm, (**c**) 17 nm, and (**d**) 18 nm Ni nanoparticles synthesized when the precursor to phosphine ratio is 1:1.2, 1:1.0, 1:0.8, and 1:0.60, respectively. (Precursor concentration = 200 mM, surfactant: TBP, aging temperature: 250°C)

Table S1. Lattice parameters of Ni nanoparticles obtained by varying precursor to phosphine ratio. The values from bulk Ni metal are also listed.

precursor : phosphine	size (nm)	(111) peak	d spacing (Å)[a]	lattice parameter (Å)[a]	(220) peak	d spacing (Å)[b]	lattice parameter (Å)[b]
1.0 : 1.2	13 nm	43.9	2.06	3.57	76.7	1.24	3.51
1.0 : 1.0	15 nm	44.0	2.05	3.56	76.5	1.24	3.52
1.0 : 0.8	17 nm	44.2	2.05	3.55	76.4	1.25	3.52
1.0 : 0.6	18 nm	44.3	2.04	3.54	76.1	1.25	3.53
Bulk		44.5	2.03	3.52	76.4	1.25	3.52

[a] The d spacing and lattice parameters are calculated on the basis of (111) peak position of XRD pattern. [b] The d spacing and lattice parameters are calculated on the basis of (220) peak position of XRD pattern.

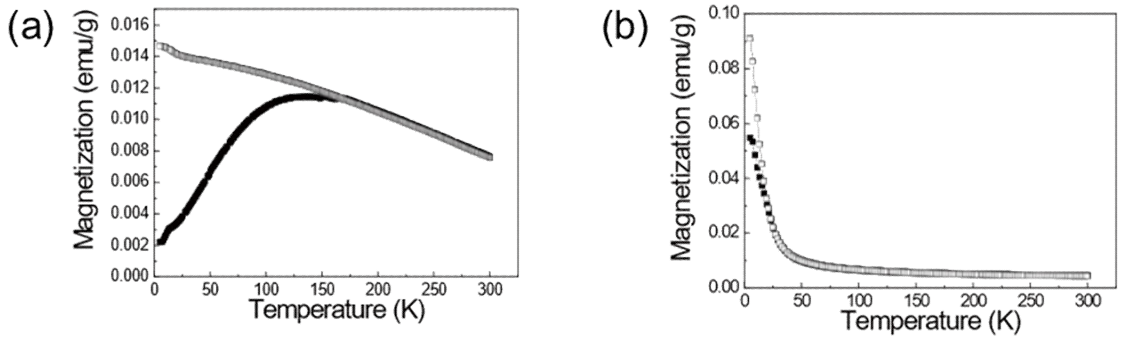

Figure S8. Temperature dependence of magnetization measured with 100 Oe for (**a**) 13 nm and (**b**) 18 nm Ni nanoparticles after zero-field cooling (■) and field cooling (□). Blocking temperature is (**a**) 19 K and (**b**) 167 K.

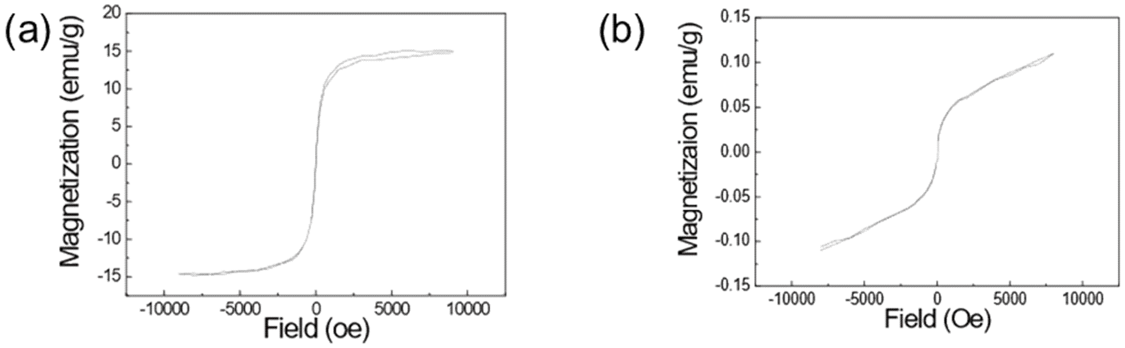

Figure S9. M-H curve of Ni nanoparticles measured by SQUID at 300 K for (**a**) 18 nm and (**b**) 13 nm Ni particles.

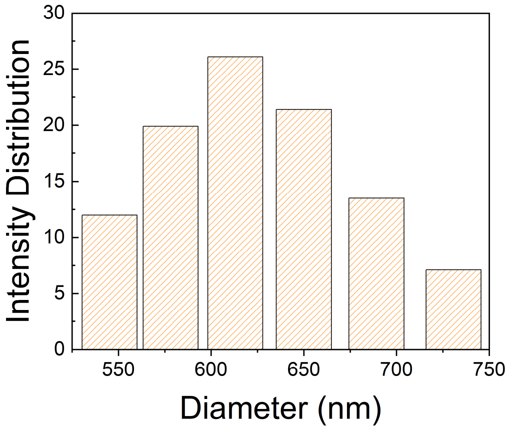

Figure S10. The hydrodynamic size distribution of nickel nanoparticles after the ligand exchange process as measured by dynamic light scattering (DLS). The average size of the ligand exchanged particles is 624.5 nm.
